# Balloon-Assisted Thrombectomy and Intrasinus Urokinase Thrombolysis for Severe Cerebral Venous Sinus Thrombosis

**DOI:** 10.3389/fneur.2021.735540

**Published:** 2021-11-18

**Authors:** Jiansheng Yang, Hongyang Wang, Yanxing Chen, Minjian Qiu, Baorong Zhang, Zhicai Chen

**Affiliations:** ^1^Department of Neurology, The Second Affiliated Hospital of Zhejiang University, School of Medicine, Hangzhou, China; ^2^Department of Ultrasonography, Affiliated Hangzhou First People's Hospital Zhejiang University School of Medicine, Hangzhou, China

**Keywords:** balloon, thrombolysis, cerebral venous sinus, thrombectomy, urokinase

## Abstract

**Background:** Current clinical guidelines recommend systemic anticoagulation as the initial treatment for severe cerebral venous sinus thrombosis (CVST). However, anticoagulation alone does not always dissolve large and extensive CVSTs in some patients. Here, we investigated the effectiveness and safety of balloon-assisted thrombectomy and intrasinus urokinase thrombolysis in our retrospective study of a series of 23 patients with CVST.

**Methods:** We reviewed the clinical, radiological, and outcome data of all patients. Complete recanalization was defined as all the occluded sinuses were recanalized on digital subtraction angiography or Contrast-enhanced magnetic resonance venography. Partial recanalization was defined as the complete recanalization of one sinus but persistent occlusion of other sinuses, or partial recanalization of one or more sinuses. The modified Rankin Scale (mRS) was used to represent the clinical outcome.

**Results:** From May 2017 to November 2019, a total of 23 patients were treated with balloon-assisted thrombectomy and intrasinus urokinase thrombolysis. A total of 84 venous sinuses were involved, ≥3 sinuses were involved in 20 (87%) patients. Among them, 21 (91%) patients achieved technical success. Complete and partial recanalization were obtained in 17 (81%) and 4 (19%) patients at 6 months follow-up, respectively. All 21 patients had mRS scores of 0 (18) or 1 (3).

**Conclusions:** Our case series shows that balloon-assisted thrombectomy combined with intrasinus urokinase thrombolysis and activated partial thromboplastin time-regulated systemic anticoagulation is safe and effective in treating severe CVST.

## Introduction

Cerebral venous sinus thrombosis is an infrequent cause of stroke, accounting for 0.5–1% of all strokes. Based on the results of some randomized controlled studies, current clinical guidelines recommend systemic anticoagulation as the initial treatment for cerebral venous sinus thrombosis (CVST) (1, 2). However, anticoagulation alone does not always dissolve large and extensive CVSTs in some patients (3). For example, heparin-based systematic anticoagulation may not be suitable for patients with antithrombin-III deficiency (4–6). Although heparin can prevent further thrombus extension, existing thrombi are not readily lysed (4, 7, 8). In CVST patients with complete occlusion in multiple sinuses, the effect of heparin is decreased because the drug cannot fully penetrate into the thrombus (9–11). According to the results of the International Study on Cerebral Vein and Dural Sinus Thrombosis, at least 13% of all patients with CVST die or remain severely handicapped (3).

Given the disadvantages of anticoagulation alone, endovascular treatment is an alternative option for patients who deteriorate despite anticoagulation (12, 13). Since the first cases published in the 1990s (14, 15), several reports have suggested that endovascular therapy is relatively safe and effective in rapidly recanalizing the thrombosed sinuses and reversing neurological deficits in severe CVST cases (9, 16, 17). Current endovascular strategies for CVST patients do not have a uniform standard. Techniques include direct catheter thrombolysis, balloon-assisted thrombectomy, mechanical thrombectomy, stent retriever thrombectomy, aspiration thrombectomy, or a combination of these. Balloon-assisted thrombectomy has the advantage of both flattening the thrombus and dilating vessels, returning previously structured vessels to their normal diameter (11, 18, 19). However, in the absence of randomized controlled trials, data about balloon-assisted thrombectomy for severe CVST patients remain insufficient. We performed a retrospective study of a series of CVST patients treated with balloon-assisted thrombectomy and intrasinus urokinase thrombolysis to investigate its effectiveness and safety.

## Methods

This study was approved by the human Ethics Committee of the Second Affiliated Hospital of Zhejiang University. The clinical investigation was conducted according to the principles expressed in the Declaration of Helsinki. Written informed consent was obtained from all patients.

Patients with CVST treated with balloon-assisted thrombectomy and intrasinus urokinase thrombolysis in our center were identified between May 2017 and November 2019. Twenty-three patients with severe CVST received endovascular treatment at our hospital. The diagnosis of CVST was based on the clinical and neurologic findings and confirmed by imaging techniques including Contrast-enhanced magnetic resonance venography (CEMRV) or digital subtraction angiography (DSA). All patients received anticoagulation with low molecular weight heparin as the initial treatment. The inclusion criteria for endovascular treatment in CVST were as follows: (1) rapid deterioration of neurological symptoms after the initiation of anticoagulation; (2) clinical presentation with lethargy or coma, venous infarction with hemorrhagic transformation, and intracerebral hemorrhage; and (3) obtained consent before the procedure. Herniation caused by large hematomas or diffuse edema were contraindications for endovascular treatment.

The procedure was performed under local anesthesia. The “Seldinger” technique was used to puncture the right femoral artery. Cerebral angiography was performed through the internal carotid artery to confirm thrombus existence and location. A 6F 90 cm vascular sheath (COOK Flexor Check-Flo Introducer, Cook Medical, Bloomington, IN, USA) was placed in the distal segment of internal jugular bulb through the right femoral vein. Then a bolus dose of heparin (2,000–3,000 U), followed by 1,000 U every hour, was intravenously administered during the procedure. A 0.014-inch microguide wire was carefully advanced through the thrombus to the distal segment beyond under microguide catheter assistance. After the catheter position was confirmed using DSA radiography, the tip of the microguide wire was kept in the distal segment beyond the thrombus, and the microcatheter was pulled out. A 4 or 5-mm rapid exchange balloon was advanced over the microguide wire to the distal segment beyond the thrombus. The balloon was gradually inflated along the thrombus segment. Then, the balloon was again advanced over the microguide wire to the distal end of the thrombus. The balloon was partially inflated and slowly drawn back to the sigmoid sinus. In some cases, the process was repeated two or three times until the partially inflated balloon could move back and forth freely from the superior sagittal sinus to the sigmoid sinus. Cerebral angiography was repeated to evaluate venous outflow pathways during the procedures.

**Figure 1:**
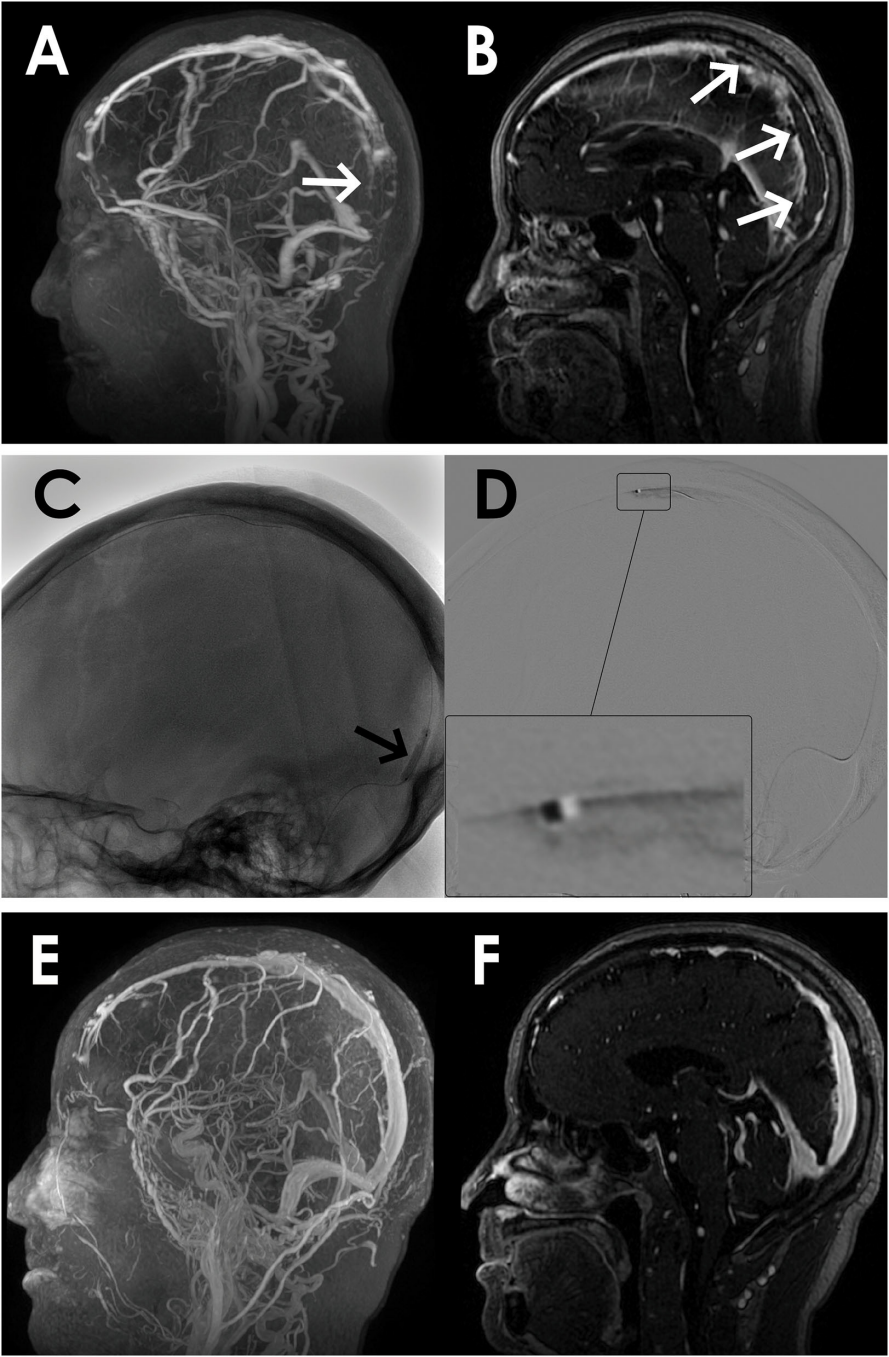
A 67-year-old man presented with acute onset of severe headache and vomiting. **(A)** Filling defects (white arrows) in the site of superior sagittal sinus on contrast-enhanced magnetic resonance venography (CEMRV). **(B)** Superior sagittal sinus thrombosis (white arrows) on source image of CEMRV. **(C)** Balloon dilatation (black arrows) in the site of superior sagittal sinus during the procedure. **(D)** Microcatheter were retained in the distal part of superior sagittal sinus for intrasinus thrombolysis by continuous injection of urokinase. **(E,F)** Complete recanalization of superior sagittal sinus on 6-month follow-up CEMRV.

After balloon dilation, the microcatheter was introduced into the distal end of the thrombus along the microguide wire. A bolus dose of 200,000-300,000 U of urokinase, followed by continuous urokinase (40,000 U/h, total 960,000 U/day) was administered into the cerebral venous sinus by microcatheter. Systemic heparinization was conducted by continuously administered heparin through the 90-cm vascular sheath to maintain an activated partial thromboplastin time (APTT) between 60 and 90 s. Follow-up DSA or CEMRV was performed 3–5 days after the procedures and before withdrawal of the heparin through the vascular sheath and urokinase through the microcatheter. The duration of intrasinus urokinase thrombolysis was based on the symptom relief and follow-up imaging findings of no more than 7 days (Figure 1). Complete recanalization was defined as all occluded sinuses were recanalized on DSA or CEMRV. Partial recanalization was defined as complete recanalization of one sinus but persistent occlusion of other sinuses, or one or more sinuses were partly recanalized. All patients subsequently started on long-term oral warfarin anticoagulation for at least 6 months, to maintain an international normalized ratio between 2 and 3.

## Results

From May 2017 to November 2019, a total of 23 patients were treated with balloon-assisted thrombectomy and intrasinus urokinase thrombolysis. The mean age was 38.0 ± 14.6 years, and 14 (61%) patients were male. The mean time from onset to diagnosis was 4 days (interquartile range, 2–34 days), and the mean duration of low molecular weight heparin anticoagulation before endovascular treatment was 8 days (interquartile range, 1–30 days). The presenting clinical characteristics are listed in Tables 1, 2. Twenty-two (96%) patients presented with headache, 10 (43%) patients with visual disturbances, and 8 (35%) patients with coma before endovascular treatment. The risk factors for CVST are shown in Tables 1, 3.

**Table 1:** Clinical baseline information of the patients with CVST.

**Case number**	**Age, sex**	**Onset to diagnosis**	**Clinical manifestation and symptoms**	**GCS**	**Comorbidities or risk factor**	**Abnormal laboratory investigation**	**Lumber puncture pressure (mm H_**2**_O)**	**Sinus involved**	**Anticoagulation before procedure**	**Recanalization at 6 month follow-up**		**Complications**	**MRS at follow-up**	**Note**
										**Partial**	**Complete**			
1	19, M	8 days	ICH, Infract, MD, coma	14	Nephrotic syndrome	WBC↑ Neutro↑ CRP↑ DD↑ Microalbuminuria↑	Non	SSS, bi-TS, StS, bi- SiS	8D	P	C	Non	0	
2	24, M	7 days	Headache, seizure, ICH, infarct, hemiparesis	15	No risk factor identified	WBC↑ Neutro↑ DD↑	400	SSS, TS, bi-SiS	7D	P	C	Non	0	
3	54, M	2 days	ICH, infarct, hemiparesis	15	Hematologic disorders/ thrombotic thrombocytopenic purpura	WBC↑ Neutro↑ DD↑ PLT↓	Non	SSS, bi-TS, StS, bi-SiS	6	C	C	Non	0	
4	48, M	7 days	Headache, infarct, hemiparesis	15	No risk factor identified	DD↑	230	SSS,TS, SIS	5	C	C	Non	0	
5	44, F	19 days	Headache	15	Hyperthyroidism/ Other risk factor	Total T3, T4↑ ESR↑ DD↑	Non	SSS, bi-TS, StS, SiS	4	P	P	Non	0	
6	27, M	7 days	Headache, seizure, MD, coma	14	Hematologic disorders	ESR↑ CRP↑ DD↑	320	SSS, bi-TS, StS, bi-SiS	2	P	P	Non	0	
7	26, M	4 days	Headache	15	Dehydration/Other risk factor	WBC↑ Neutro↑ DD↑ CRP↑	400	SSS, TS, SiS	5	P	C	Non	0	
8	44, F	5 days	Headache, seizure, ICH, infarct, hemiparesis	15	Oral contraceptives	WBC↑ Neutro↑ DD↑ CRP↑ PLT↓	400	SSS, TS, SiS	5	P	P	Non	0	Relapsed
9	35, M	15 days	Headache, infarct	15	No risk factor identified	DD↑	Non	SSS,TS, SiS	30	P	P	Non	0	Technical failure
10	58, M	6 months	Headache, VD	15	Hyperhomo cysteinemia/ Coagulation disorders	WBC↑ Neutro↑ DD↑ CRP↑ PLT↑ ESR↑ Homocysteine↑	260	SSS, TS, SiS	10	P	P	Non	0	Technical failure
11	35, F	1 day	Headache, VD	15	Hematologic disorders/ anemia	Hb↓ RBC↓DD↑	400	SSS, StS, TS, SiS	5	P	P	Non	0	
12	67, M	5 days	Headache	15	No risk factor identified	TG↑ LDL↑ HDL↓DD↑	265	SSS,TS, SiS	10	C	C	Non	0	
13	63, M	2 days	Headache, seizure, ICH, infarct, hemiparesis, coma	10	Nephrotic syndrome	Hb↓ RBC↓DD↑ WBC↑ Neutro↑ CRP↑ proteinuria↑	220	SSS, StS, TS, SiS	1	C	C	Non	1	
14	22, M	7 days	Headache, VD, hemiparesis	15	Nephrotic syndrome	Hb↓ RBC↓DD↑ WBC↑ Neutro↑ CRP↑ proteinuria↑	400	SSS, StS, TS, SiS	4	C	C	Non	0	
15	25, M	3 months	Headache, VD	15	Dehydration/Other risk factor	DD↑ proteinuria↑	300	StS, SiS	30	P	C	Non	1	Blurred vision did not improve
16	31, F	5 days	Headache, MD, coma	12	Oral contraceptives	Neutro↑ DD↑ Homocysteine↑ Total T4↑ TG↑ LDL↑ HDL↓	360	SSS, StS, TS, SiS	1	P	C	Non	0	
17	31, F	15 days	Headache, hemiparesis, VD, MD	15	With history of craniotomy./ Other risk factor	Neutro↑ DD↑	120	SSS, TS, SiS	1	P	C	Non	0	
18	49, F	5 days	Headache, hemiparesis, VD	15	Protein C and S deficiency/ Coagulation disorders	CRP↑ DD↑ Hb↓PLT↑ PS↓ PC↓	Non	TS, StS, SiS	6	P	C	Non	0	
19	56, M	4 days	Headache, seizure, ICH, coma	14	Hyperhomo cysteinemia/ Coagulation disorders	DD↑ CRP↑ Neutro↑ Homocysteine↑	Non	SSS, TS, SiS	1	C	C	Non	0	
20	47, M	3 days	Headache, seizure, VD, coma	14	Protein C and S deficiency/ Coagulation disorders	WBC↑ Neutro↑ CRP↑ DD↑ PS↓ PC↓LDL↑	320	SSS, TS, SiS	1	C	C	Non	0	
21	20, F	10 days	Headache, VD, coma	14	Oral contraceptives	WBC↑ Neutro↑ CRP↑ DD↑ Total T4↑	400	SSS, bi-TS, bi-SiS	1	P	C	Non	0	
22	24, F	2 days	Headache, ICH, hemiparesis, VD, MD, coma	14	Pregnancy or puerperium	WBC↑ Neutro↑ CRP↑ DD↑ Hb↓PLT↑	Non	TS, SiS	1	P	C	Hematoma enlargement	1	
23	24, F	7 days	Headache, seizure, ICH, infarct, hemiparesis, VD	15	Pregnancy or puerperium	WBC↑ Neutro↑ CRP↑ DD↑ PLT↑	Non	SSS, StS	1	C	C	Non	0	

*CVST, cerebral venous sinus thrombosis, ICH, Intracranial hemorrhage; MD, Mental status disorder; VD, Visual disturbances; SSS, superior sagittal sinus; SiS, sigmoid sinus; StS, straight sinus; TS, transverse sinus; F, Female; M, male; WBC, white blood cell; Neutro, Neutrophil; PLT, platelet; Hb, hemoglobin; RBC, red blood cell; CRP, C-reactive protein; DD, d-dimer; PS, Protein S; PC, Protein C; T3, triiodothyronine; T4, Thyroxine;ESR, erythrocyte sedimentation rate; TG, triglycerides; LDL, Low-density lipoprotein cholesterol; HDL, High-density lipoprotein cholesterol; Non, none. ↑, higher than upper reference level; ↓, lower than lower reference level*.

**Table 2:** Patient characteristics and clinical presentation in 23 patients with severe cerebral venous sinus thrombosis (CVST).

**Characteristic**	**Number of patients**
Patient characteristics	
Male	14
Mean age (years ± SD)	38 ± 14.6
Anticoagulation before procedure (days and interquartile range)	8 (1–30)
Clinical presentation	
Headache	22 (96%)
Visual disturbances	10 (43%)
Hemiparesis	10 (43%)
Infarct	9 (39%)
Intracranial hemorrhage	8 (35%)
Coma	8 (35%)
Seizures	7 (30%)
Mental status disorder	5 (21%)

**Table 3:** Risk factors in 23 patients with severe CVST.

**Cause or risk factors**	**Number of patients**
Nephrotic syndrome	3
Pregnancy or puerperium	2
Oral contraceptives	3
Coagulation disorders: protein C deficiency, protein S deficiency, hyperhomocysteinemia	4
Hematologic disorders: anemia, thrombotic thrombocytopenic purpura	3
Other risk factor	4
No risk factor identified	4

A total of 84 venous sinuses were involved in 23 patients, ≥3 sinuses were involved in 20 (87%) patients. The transverse sinus (96%) and sigmoid sinus (96%) were the most affected (Table 4). Overall, 21 (91%) patients achieved technical success and 2 patients failed because the microguide wire could not penetrate the hard thrombus into the true lumen. One patient deteriorated due to hematoma enlargement while undergoing intrasinus thrombolysis and intravenous anticoagulation. This patient was switched to oral warfarin anticoagulation, and their symptoms completely resolved following hospital discharge.

**Table 4:** Dural sinus involvement in patients with CVST.

**Location of thrombus**	**Number of patients (%)**
Superior sagittal sinus	20 (87%)
Transverse sinus	22 (96%)
Bilateral transverse sinus	6 (26%)
Sigmoid sinus	22 (96%)
Bilateral sigmoid sinus	4 (17%)
Straight sinus	10 (43%)
Involved sinuses ≥3	20 (87%)

Complete and partial recanalization was obtained in 8 (38.1%) and 13 (61.9%) patients at discharge, respectively. All patients had symptom relief upon discharge. One patient with visual disturbances at admission still had blurred vision, and two patients with hemiparesis at admission had left limb dysfunction at discharge. All patients were followed up for at least 6 months. Complete and partial recanalization were confirmed in 17 (81.0%) and 4 (19.0%) patients (Table 5), respectively. All 21 patients had modified Rankin scale (mRS) scores of 0 (18 patients) or 1 (3 patients). The patient with blurred vision at discharge did not improve further, but both patients with limb dysfunction at discharge improved slightly during the follow-up period. One patient who had recanalized sinuses and complete recovery at discharge relapsed because of self-discontinuation of warfarin. After intravenous anticoagulation and bridging oral warfarin therapy, the patient was discharged with improved symptoms and there was no recurrence during follow-up.

**Table 5:** Recanalization rate at discharge and 6-month follow-up.

	**Partial recanalization**	**Complete recanalization**
Discharged	13 (61.9%)	8 (38.1%)
Follow-up	4 (19.0%)	17 (81.0%)

## Discussion

Although the incidence of CVST is low, early identification and appropriate treatment are crucial for good patient prognosis (20, 21). Heparin or low molecular weight heparin is currently the first-line treatment for CVST. However, despite these medications, 13.6% of patients have poor outcomes with an 8.3% mortality rate after an average follow-up of 16 months (3, 22). Patients with more severe clinical presentations (e.g., coma, mental status disorder, intracranial hemorrhage on admission) and larger intracranial thrombus burden tend to have worse prognoses (12, 23). The management of severe CVST is still challenging, with a mortality rate as high as 34.2% (3, 21, 24), suggesting that more aggressive treatment strategies are needed.

US guidelines recommend endovascular treatment as an option for patients who are comatose or deteriorate despite anticoagulation (2, 25). Endovascular procedures including direct catheter thrombolysis and mechanical thrombectomy may better recanalize occluded venous sinus and subsequently relieve related symptoms (10). Previous studies using different endovascular techniques highlighted the effectiveness and safety of endovascular treatment (9, 12, 15, 16, 26–28). A systematic analysis conducted by Ilyas et al. enrolled 235 patients with CVST treated by mechanical thrombectomy combining catheter thrombolysis and found that 69% of patients were completely recanalized (12). In another systematic review of direct catheter thrombolysis for 169 patients with CVST, 48% patients obtained completely recanalization after catheter thrombolysis alone (20). This suggests that the combination of direct catheter thrombolysis and mechanical thrombectomy may improve the prognosis better than a single technique. In the current study, we employed endovascular treatment combining balloon-assisted thrombectomy and intrasinus urokinase thrombolysis. Overall, 38.1 and 81% patients obtained complete recanalization at discharge and at 6-month follow-up, respectively. This recanalization rate is similar to previous studies combining endovascular thrombectomy and thrombolysis. In addition, all 23 patients had symptom relief at discharge and good outcomes at the 6-month follow-up.

Previous studies mainly focused on stent retrievers or aspiration devices, with little attention paid to balloon thrombectomy and angioplasty (12, 26). The balloon-assisted thrombectomy techniques used in our study included balloon angioplasty and dragging a partially inflated balloon forward and backward like a shuttle. Both approaches can help loosen and break down the thrombus, increase the contact area between the thrombus and urokinase, and restore the venous outflow channel. Besides being convenient and cheap, angioplasty with a balloon catheter has the advantages of both loosening the thrombus and simultaneously dilating vessels to a normal diameter (11, 19). There were no operation-related complications in our case series, which is consistent with the results reported by Shui et al. (11). In their previous study which investigated balloon-assisted thrombectomy alone to treat patients with CVST, Shui et al. extracted thrombi using balloon dilatation and drawing, and the results showed it was safe and effective. Therefore, we propose that balloon-assisted thrombectomy combined with intrasinus urokinase thrombolysis can be considered as an alternative treatment for CVST.

It remains controversial whether thrombolysis could be used in patients with CVST with intracerebral hemorrhage (16, 20, 29, 30). Some clinicians are concerned that thrombolysis may enlarge the hematoma and potentially lead to poor prognosis (29, 30). Others portend that thrombolysis can reduce the thrombus burden, thus improving the hematoma and prognosis. In particular, the dosage of intrasinus thrombolytic drugs is relatively small compared to systemic thrombolysis (12, 16, 31). In our case series, we used intravenous thrombolysis and systemic anticoagulation based on APTT monitoring and demonstrated that balloon-assist thrombectomy was safe and effective in stopping neurological deterioration (9). Only one patient deteriorated due to hematoma enlargement while undergoing intrasinus urokinase thrombolysis and intravenous anticoagulation. The condition of the patient was stable and completely recovered at discharge after halting intrasinus thrombolysis and intravenous anticoagulation and switching to oral warfarin.

Thrombus location, extension, and composition all affect the success of endovascular treatment (13). In our study, two patients with chronic CVST had well-organized thrombus formation, which was the main cause of procedure failure. In the successful group, a total of 78 venous sinuses were involved in 21 patients. Among them, 18 patients had ≥3 sinus involvement. We found that 4 (19%) patients did not achieve complete recanalization even at the 6-month follow-up, especially when several venous sinuses were involved at the same time. Our results demonstrate that multiple thrombus is a risk factor of poor recanalization, which is similar to previous studies (3, 13). However, it should be noted that there is no direct correlation between complete recanalization and clinical outcome (9, 32). The rate of good outcome at discharge was 100% in our study, although the complete recanalization rate at that time was lower than that at 6-month follow-up. This phenomenon suggests that patients with CVST can achieve good outcomes as long as they achieve partial recanalization.

## Conclusion

Our case series shows that balloon-assisted thrombectomy combined with intrasinus urokinase thrombolysis and Systemic anticoagulation base on APTT monitoring is safe and effective for patients with severe CVST. Given the limitations of a retrospective design and small sample, a randomized controlled trial is needed to confirm our findings.

## Data Availability Statement

The original contributions presented in the study are included in the article/supplementary material, further inquiries can be directed to the corresponding author/s.

## Ethics Statement

The studies involving human participants were reviewed and approved by Human Ethics Committee of the Second Affiliated Hospital of Zhejiang University. The patients/participants provided their written informed consent to participate in this study.

## Author Contributions

JY and HW wrote the manuscript. YC and MQ wrote and edited the manuscript. BZ and ZC reviewed the manuscript. All authors approved the final version.

## Conflict of Interest

The authors declare that the research was conducted in the absence of any commercial or financial relationships that could be construed as a potential conflict of interest.

## Publisher's Note

All claims expressed in this article are solely those of the authors and do not necessarily represent those of their affiliated organizations, or those of the publisher, the editors and the reviewers. Any product that may be evaluated in this article, or claim that may be made by its manufacturer, is not guaranteed or endorsed by the publisher.
